# Eye movements reveal the contributions of early and late processes of enhancement and suppression to the guidance of visual search

**DOI:** 10.3758/s13414-022-02536-w

**Published:** 2022-07-20

**Authors:** Zachary Hamblin-Frohman, Seah Chang, Howard Egeth, Stefanie I. Becker

**Affiliations:** 1 School of Psychology, University of Queensland, Brisbane, QLD Australia; 2 Department of Psychological and Brain Sciences, Johns Hopkins University, Baltimore, MD USA

**Keywords:** Visual search, Attention, Inhibition, Enhancement, Suppression

## Abstract

In visual search attention can be directed towards items matching top-down goals, but this must compete with factors such as salience that can capture attention. However, under some circumstances it appears that attention can avoid known distractor features. Chang and Egeth (Psychological Science, 30 (12), 1724–1732, 2019) found that such inhibitory effects reflect a combination of distractor-feature suppression and target-feature enhancement. In the present study (*N* = 48), we extend these findings by revealing that suppression and enhancement effects guide overt attention. On search trials (75% of trials) participants searched for a diamond shape among several other shapes. On half of the search trials all objects were the same colour (e.g., green) and on the other half of the search trials one of the non-target shapes appeared in a different colour (e.g., red). On interleaved probe trials (25% of trials), subjects were presented with four ovals. One of the ovals was in either the colour of the target or the colour of the distractor from the search trials. The other three ovals were on neutral colours. Critically, we found that attention was overtly captured by target colours and avoided distractor colours when they were viewed in a background of neutral colours. In addition, we provided a time course of attentional control. Within visual search tasks we observed inhibition aiding early attentional effects, indexed by the time it took gaze to first reach the target, as well as later decision-making processes indexed by the time for a decision to be made once the target as found.

## Introduction

Visual search requires an individual to direct attention throughout an array of stimuli to locate a target item. Human behaviour often makes use of efficient strategies to perform tasks, and this extends to the visual search paradigm. One such strategy is to bias the visual system to features that match the defining aspect of the target in a top-down controlled manner (e.g., Desimone & Duncan, 1995; Treisman & Gelade, 1980). Top-down tuning to the target, or target-feature enhancement, is a mechanism that drives attentional allocation to feature matching stimuli (e.g., Bichot et al., 2005; Harris et al., 2013). However, target-feature enhancement does not necessarily lead to perfect selection of the search target. Contingent capture effects are observed when irrelevant items share the target’s defining feature, causing attention to be directed towards these items, resulting in task performance deficits (e.g., Folk et al., 1992). These effects highlight that biasing the attentional system to prioritise features matching the target is one of the key mechanisms used in search guidance.

Conversely, items not related to target-feature information can sometimes attract attention independently of current intentions in a purely stimulus-driven, bottom-up manner. Non-target items that are saliently different from their environment (such as abrupt onsets, moving items or items with a unique colour) can attract attention, resulting in slower response times (RTs) and less efficient search (e.g., Becker, 2007; Theeuwes et al., 1998). Stimulus-driven capture has been proposed to be due to the inherent saliency of singleton items compared to their surroundings (Theeuwes, 1992). A feature singleton capturing attention makes inherent sense; an item that varies greatly from its surrounding may often be indicative of information relevant to behaviour. However, multiple studies have reported that unique items, or distractors, do not always capture attention. Folk et al. (1992) and Bacon and Egeth (1994), among others, have shown the importance of the attentional set in determining whether capture occurs or not (e.g., when subjects are set to search for a colour target, a sudden-onset distractor may not capture attention). Such results are referred to as contingent capture and have been taken to support a goal-driven, as opposed to stimulus-driven, account of attentional capture.

Inhibition is a proposal to reconcile the stimulus-driven and goal-driven accounts. It has been found that there are circumstances in which previous exposure to an irrelevant distractor feature can result in the suppression of the distractor, which limits its ability to attract attention and helps to guide behaviour in a goal-directed manner. Several paradigms have found evidence for distractor suppression, from behavioural studies where an informative cue about an upcoming distractor was able, with practice, to reduce capture (e.g., Cunningham & Egeth, 2016), to electrophysiological studies showing neural responses associated with feature suppression when distractors are presented (Gaspelin & Luck, 2018; Moher et al., 2014). It is assumed that while salient stimuli can produce a priority signal, in accordance with the stimulus-driven models, attentional capture can be prevented by an inhibitory process that suppresses the known distractor features, in accord with goal-driven models. For a more complete view of the 25-year debate between adherents of the stimulus-driven and goal-driven theories of attentional capture, including the attempted reconciliation of the two approaches, see Luck et al. (2021).

It should be noted that it is still unclear whether inhibition is due to an active/top-down or passive/automatic mechanism (e.g., Becker, 2007; Gaspelin & Luck, 2018; Kerzel & Burra, 2020). Inhibition of a distractor is likely mediated by inhibition of the particular feature value associated with the distractor (e.g., colour, position; Ruthruff et al., 2021; Treisman & Sato, 1990). It has been proposed that we may be able to suppress any salient signal, irrespective of the feature (Sawaki & Luck, 2010). However, the proponents of this view now emphasize a feature-driven inhibition view (e.g., Gaspelin & Luck, 2019). Hence, we will often use the term ‘feature inhibition’ to refer to inhibition of the distractor but will remain agnostic about whether it is an active or passive mechanism.

One of the most straightforward depictions of inhibition comes from the visual search literature. Gaspelin et al. (2015) used a capture-probe paradigm to assess attentional capture (or lack thereof) as a measure of the inhibitory response to a singleton distractor stimulus. Participants completed a visual search for a shape-defined stimulus in a shape-heterogeneous array. On 50% of trials, all items (target and non-targets) were a consistent colour (e.g., green). For the other 50%, a distractor item with a unique colour (e.g., red) replaced one of the non-targets, the target/non-target and distractor colours remained consistent throughout the experiment. The authors found, after participants had practiced the search in a warm-up block, that RTs were quicker on distractor-present trials over distractor-absent trials, implying that the distractor was not just ignored but had been *inhibited*. Further studies using eye movements revealed that the first saccade in search is less likely to land on the distractor than one of the non-target items, further suggesting an attentional bias away from the distractor feature (Gaspelin et al., 2017). A third measure of inhibition came from memory probe trials used by Gaspelin et al. (2015). The memory probe displays resembled the search displays but were presented only briefly, and observers were asked to recall characters contained within the stimuli. Results revealed that memory accuracy was higher for probes matching the target colours from the search trials than those that matched the distractor colour from the search trials (Gaspelin et al., 2015). These search and probe trial results indicate that a mechanism is in place that allows suppression of distractor features, promoting efficient search.

A recurring issue with studies investigating inhibition via visual search is that the target-related and distractor-related colour features are often presented in direct competition with each other (e.g., Gaspelin et al., 2015). Potentially, instead of distractor feature suppression, results that look like inhibition could instead be due to target-feature enhancement. In previous inhibition studies all of the non-target items (not including the distractor) had the same colour as the target item, in both probe and search trials. For instance, participants had to search for a green diamond among green non-target shapes and ignore an irrelevant red item (Gaspelin et al., 2015). Thus, it is unclear if attentional biasing effects were driven by inhibiting the distractor colour (red) or biasing attention to the target colour (green). As the non-targets had the same colour as the target, both potential explanations could explain the finding that attention and eye movements were biased away from the distractor feature, accounting for RT and memory recall differences.

A secondary concern with the probe trials is that using a memory-based paradigm may involve additional memory-related processes besides attentional capture by the stimuli. Recall may have been decreased towards the distractor items due to a lower prioritisation of the contained character, for example, the character contained in the distractor was reported last and thus less likely to be recalled correctly. This leaves the possibility that inhibitory effects found on the probe trials may not be due to early attentional biasing but instead due to later memory effects.

Chang and Egeth (2019, 2021) addressed these concerns by disentangling the unique effects of target enhancement and distractor suppression and modifying memory probe trials to tap into earlier attentional processes. To that end, Chang and Egeth (2019) changed the probe trials to become a forced-choice task. One of the probes contained the letter A or B, and subjects were asked to report which was present with a button press. As this now required a single forced-choice response, the potential memory-related problems were mitigated. Secondly, the memory probe displays were modified to allow for the separate effects of target-feature enhancement and distractor-feature suppression to be examined.

Specifically, the probe trials were divided into two equal-sized, randomly intermixed sets. One set had a target-coloured item presented among neutral-coloured items (i.e., colours not used in the search task). The other set had a distractor-coloured item presented among neutral-coloured items. The authors found that attention was drawn towards the target colour probe compared to the neutral colours, reflecting target-feature enhancement, and that attention was also biased away from the distractor-colour probe compared to neutral colours, reflecting distractor inhibition (measured via both response times and probe accuracy; Chang & Egeth, 2019). These results led to the conclusion that both distractor-feature suppression *and* target-feature enhancement contributed to the original effects observed in the capture-probe paradigm.

The current research aimed to expand on the previous work of Chang and Egeth (2019) by examining the course of eye movements during visual search and probe trials. Previous research has shown that eye movements avoid repeated consistent distractor features (e.g. Gaspelin et al., 2017); however, this has not been examined when disentangling target versus distractor-related effects. Furthermore, while Chang and Egeth (2019) collected RTs and accuracy rates, it is possible the observed biases were not due to early attentional guidance but instead a later-stage effect, such as a response-based bias or other decision processes commencing after the target had been located. Accuracy and RT data can reflect additional behavioural constructs beyond just attentional allocation. For example, conscious decision making and perceptual biases can influence RTs and accuracies on probe trials (see Hamblin-Frohman & Becker, 2021). On inhibition search trials, participants responded to the target stimulus and did not respond to the distractor-coloured stimulus. This repetition of responding or ignoring may have influenced responses towards these stimuli in the probe trials, superseding any attentional benefits. Eye-movement data will reveal if there are attentional biases towards the target and/or away from the distractor features by inspecting first-saccade capture. Distractor-feature suppression should be reflected in a reduced likelihood of selecting the distractor-coloured matching probes with the first eye movement compared to neutral-coloured probes, whereas target-feature enhancement should be reflected in an increased likelihood of selecting target-feature matching probes.

A second aim of this study was to assess the relative contributions of early and late processes of enhancement and suppression to the guidance of search. If these effects purely influence early attention-guiding processes then there should be the aforementioned avoidance of the distractor (colour) and the facilitation of target localisation. In addition, if inhibition of the distractor colour can also affect later, decision or response-related processes, then the inhibitory effects should be seen at the decision-making phase of the trial, after the target has been located. In the present study, we separated early- versus late-stage processes by reporting effects separately for the time from the onset of the display to the first eye movement to the target (early) and from the first fixation on the target to the keyboard response (late).

It should be noted that the time to the onset of the first eye movement to the target can potentially include later processes unrelated to guidance as well, such as when a non-target or the distractor is selected prior to the target. In this instance, the time required to select the target will include the dwell times on the irrelevant item, which can include post-selective processes related to non-target identification or decision-making (e.g., non-target rejection; e.g., Becker, 2011; Duncan, 1980; Martin & Becker, 2018). To obtain a more refined measure for early and later processes we analysed the proportion of first eye movements to each item type (target, distractor, non-target), which should only reflect early, attention-guiding processes, and separately the dwell-times for each item. Dwell times on the non-target and distractor prior to target selection were classified as belonging to an ‘intermediate’ stage, as they influence the speed of target selection but are themselves conceivably influenced by post-selective processes (e.g., decision-making). Target dwell times reflect processes that commence after selection of the target and are not related to attentional guidance or selection, and are therefore classified as reflecting late-stage processes. Overall, this separation into early (very early and intermediate) versus late measures in the eye-movement data should lead to new insights that help to map out the functional benefits of distractor suppression and target enhancement in visual search.

## Methods

### Participants

To estimate the required sample size, we used the smallest observed effect in the study of Chang and Egeth (2019), which was the RT difference between distractor-present and -absent trials; *t*(59) = 3.90. To achieve a power of 95% (with 50% assurance) the BUCSS tool (Anderson et al., 2017) suggested a sample size of 55 participants.

Fifty-nine paid participants at the University of Queensland participated in the experiment. Eight participants were excluded because of eye-tracking failures (< 25% of trials recorded eye movements). Three participants were excluded as their overall number of analysable trials was below 50% (see *Results* for trial exclusion criteria). This left 48 participants (*mean* age = 23.6 years, 42 female) for the final analysis. As the sample size still yielded adequate power (> 90%; to achieve a power of 90% would have required 48 participants according to the BUCCS tool), we did not collect additional data after the exclusions were made. All participants reported normal or corrected-to-normal vision. Study approval was granted by the University of Queensland’s Faculty of Psychology Ethics Board.

### Apparatus

Stimuli were presented on a 21-in. CRT monitor (refresh: 60 Hz). A chin and headrest were used to hold the participant’s head in a constant position 60 cm from the screen. Gaze location was measured by an SR-Research Eyelink-1000 eye tracker at a 500-Hz sampling rate. The experiment was controlled by PsychoPy in Python language (Peirce, 2007).

### Stimuli

Stimuli were presented against a white background. Throughout all trials a fixation cross was drawn at the centre of the screen. The search and probe stimuli were presented in a diamond configuration, with each element being 6.68° of visual angle away from the fixation mark. This marked an increase in distance from Chang and Egeth (2019), the purpose being to increase the need for eye movements within the trial. Overall, there were several differences in the display and stimuli from the previous work, designed to increase the sensitivity of the probe trials and tailor the stimuli for the eye-tracking paradigm.

The search stimuli consisted of four different shapes: the target diamond (1.43° x 1.43°), a square (1.43° x 1.43°), a circle (diameter: 1.72°) and a hexagon (height: 2.00°, width: 1.43°). Within each of the search shapes was a small ‘x’ or ‘+’ (height: 0.48°) that served as the response-related items and were kept small to encourage participants to make saccades to the centre of the stimuli (and avoid saccadic undershoot; e.g., Findlay et al., 1993). The probe stimuli were all ovals (height: 1.91°, width: 2.48°). Each probe contained a character, 0.57° in height. The critical (target) probe contained a numeral (2 through 9) while the three non-target probes contained an uppercase letter (A, C, F, K, M, R, V, W, Y). We used a numeral among letters as the target in the probe trials because numerals can be quickly distinguished from letters, allowing participants to quickly find the target on probe trials; and the numerals allowed the use of different numerals (2–9), which reduced chance probability and rendered the task more sensitive. Five equiluminant (30 ± 2 cd/m^2^) colours were used in the experiment: red, gold, green, blue and purple. Each participant was randomly assigned one of the colours as the search target and another as the distractor. The other colours were used as the neutral colours in the probe trials.

### Design and procedure

**Fig. 1:**
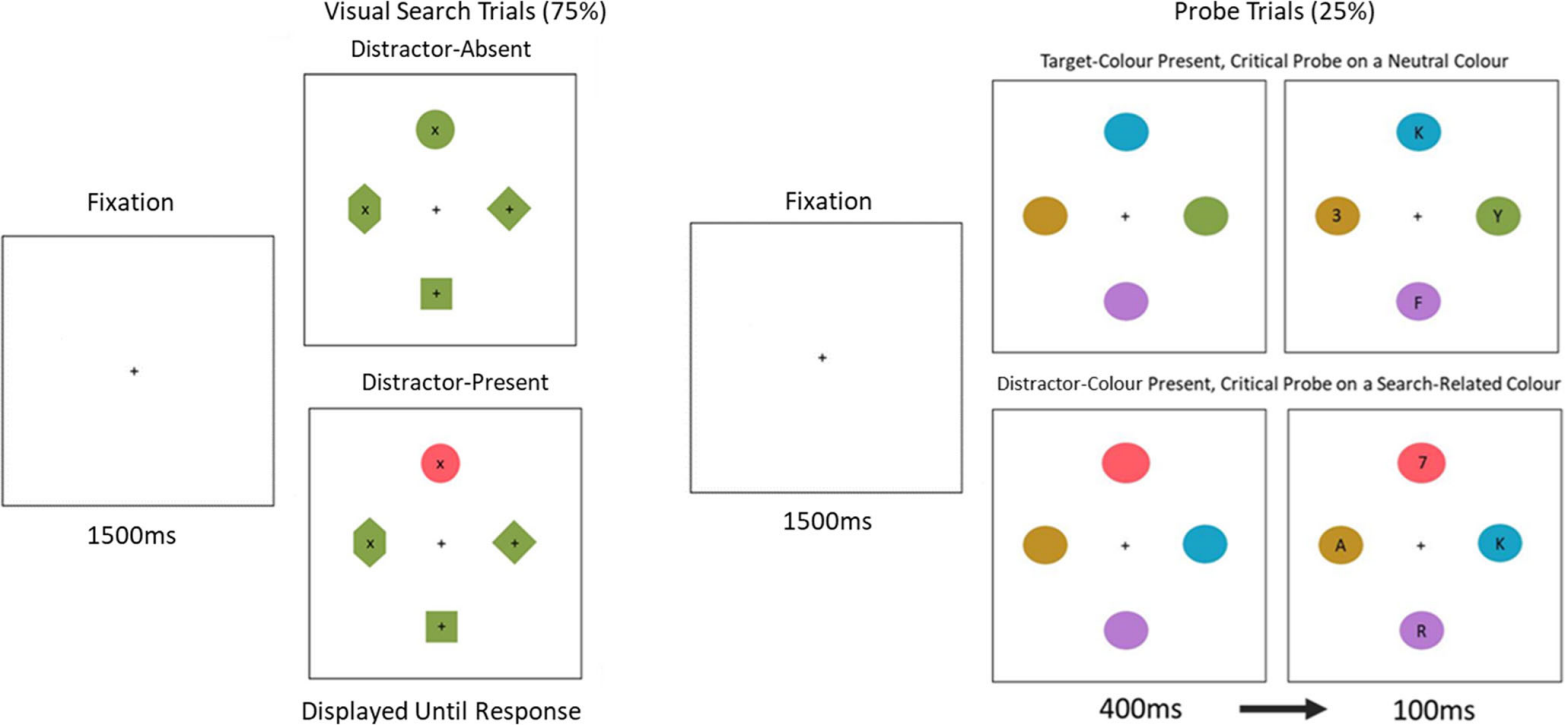
**Left:** Visual search trials (75%) were displayed until participants made a response to the character contained within the target-diamond shape. The target and the non-target items retained a consistent colour throughout the experiment (e.g., green). On 50% of trials one of the non-target items was coloured as the distractor (e.g., red). **Right:** Probe trials appeared on 25% of trials. On half of the probe trials one of the probes was target-colour matching (e.g., green) and on the other half distractor-colour matching (e.g., red), while the other three probes were unrelated to the visual search trials (referred to as *neutral probes*). Participants responded to a briefly displayed numeral (referred to as the *critical probe*), which was either located on a search-related colour (i.e., matching either the target or distractor colour) or on a neutral item

In the main block of the experiment, participants completed 480 trials with search (75%) and probe (25%) trials randomly intermixed. Each trial began with participants maintaining fixation for 1,500 ms, after which the task screen was presented. For the search trials the stimuli were displayed until the response (see Fig. 1 for a depiction of the search trials). Participants were instructed to respond to the character contained within the diamond shape with the ‘x’ key if it was an x or the ‘p’ key if was a +. The distractor was presented on 50% of search trials, and participants were informed that the target diamond would never be in the distractor colour. If participants responded later than 1,500 ms a feedback message was displayed after the response was made reading “Too Slow!” Participants were not given specific instructions on how to move their eyes during the trial, but eye movements were behaviourally encouraged via the large distance of items from fixation and the reduced character size.

On the probe trials the presentation of the characters was delayed: the coloured probe ovals were first displayed for 400 ms after which alphanumeric characters were added to the screen for an additional 100 ms. This allowed initial attentional selection based on the probe colours to be observed via eye movements (see Fig. 1). Three of the characters contained a letter and the other a numeral (the critical probe). Participants were instructed to report what the numeral was (using the corresponding numeric keys). Of the probes, three were neutrally coloured (colours not used in the visual search) and one matched either the colour of the target from the search trials (referred to as a target-coloured probe) or, equally likely, matched the colour of the distractor from the search trials (a distractor-coloured probe). We use the labels of target and distractor colours in the probe trials, but it should be noted that these descriptions refer to their roles in the visual search trials. All probes were equally likely to contain the critical numeral, that is, on 25% of trials the critical probe was on the visual search target or distractor colour (12.5% on each), and on 75% of trials it was on a neutral colour. Before the main block of the experiment participants completed 96 practice search trials (these were not included in the main analysis), followed by eight practice probe trials, on which trials the ovals were all grey.

### Eye movement data

Eye movements were parsed into saccades, fixations and blinks using the standard parser configuration of the Eyelink software, which classifies an eye movement as a saccade when it exceeds a velocity of 30°/s or an acceleration of 8,000°/s. Fixations were assigned to the target, a non-target or the distractor when the gaze was within 2.5° of visual angle from the centre of the stimulus.

## Results

### Search trials

Trials were discarded based on four criteria. Search trials were excluded when: responses were longer than 1,500 ms (8.1% of trials); responses were incorrect (11.7%); first saccade latencies started later than 1,000 ms or earlier than 100 ms (5.6%); and when eyes did not leave fixation (11.25° away from the fixation cross; < 1% of trials). This led to an average of 74.6% usable trials per participant.

#### Mean RT

Replicating previous inhibition studies a ~20-ms RT benefit was observed in distractor-present trials (*M* = 973.2 ms) compared to distractor-absent trials (*M* = 993.0 ms), *t*(47) = 6.17, *p* < .001, 95% CI [13.3, 26.4]. Search accuracy did not significantly differ between absent (*M* = 86.5%) and present (*M*= 87.4%) trials (*p* = .085).

#### First eye movements

**Fig. 2:**
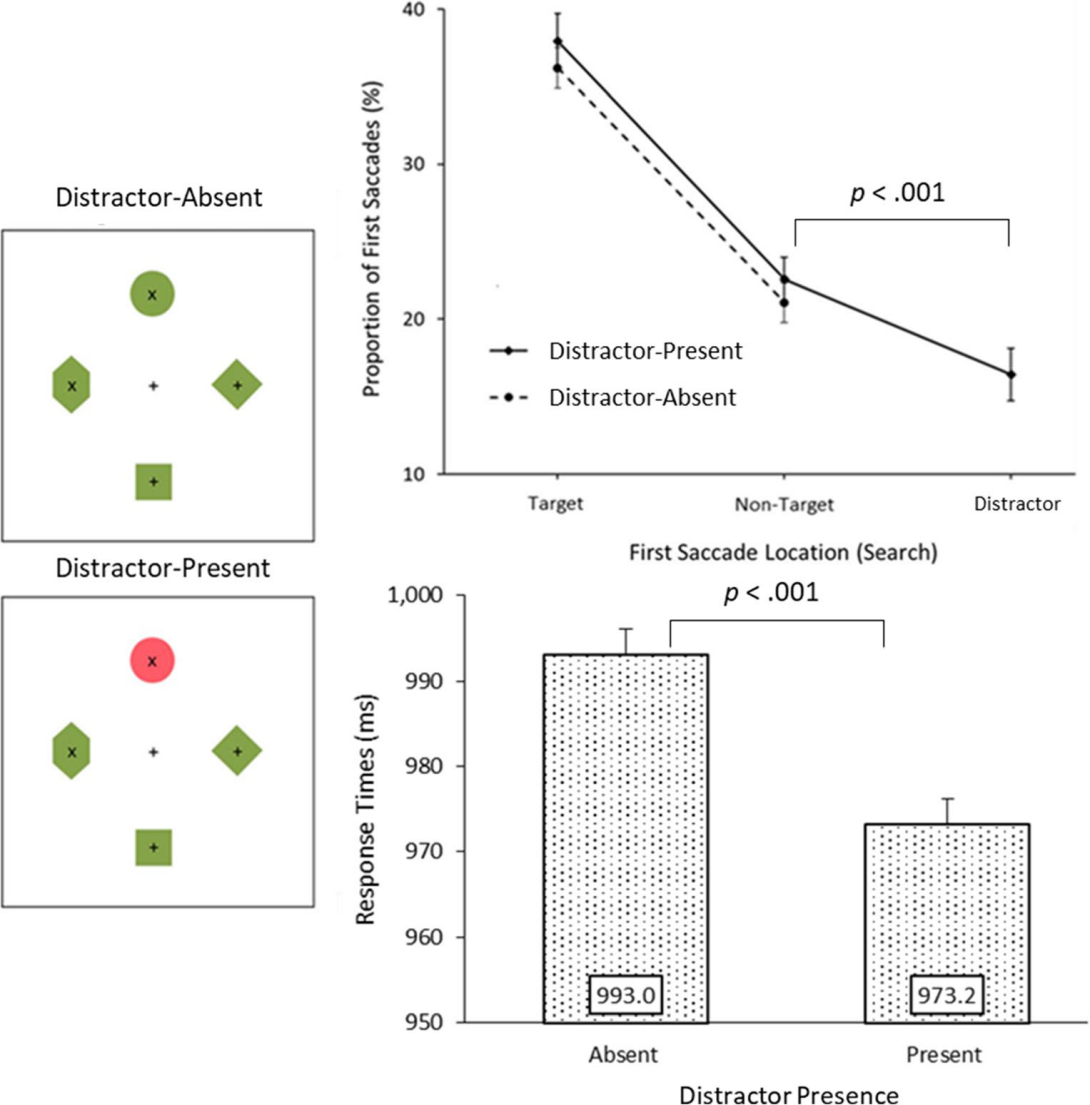
Results from the visual search trials. **Above:** The proportions of first saccades directed to the target, average non-target item and distractor. Importantly on distractor-present trials saccades were less frequently directed towards the distractor than towards the non-target items. **Below:** Search response times; the target was responded to quicker when the distractor was present compared to absent. Error bars represent within-subject 95% confidence intervals (Loftus & Masson, 1994)

To gauge whether distractor presence affected early attentional processes, we first conducted a one-way repeated-measures ANOVA, on distractor-present search trials, over the proportions of first saccades to target, non-target, and distractor items. Saccades to the non-target items are reported as the average proportion of first saccades directed to a single non-target (i.e., total proportion of first eye movements landing on all non-targets divided by the total number of non-targets present for that trial type; see Fig. 2). Results revealed a significant effect of first saccade location, *F*(2,94) = 126.84, *p* < .001, ƞ^2^_p_ = 0.73. Paired-sample t-tests revealed that the distractor (*M* = 16.4%) was selected less frequently than both the non-targets (*M* = 22.6%), *t*(47) = 5.35, *p* < .001, 95% CI [3.8, 8.5] and the target (*M* = 37.9%). The targets were fixated significantly more frequently the non-targets, *t*(47) = 11.03, *p* < .001 95% CI [12.6, 18.2].

There were more non-target first fixations on distractor-present (*M* = 22.6%) trials than -absent trials (*M* = 21.1%), *t*(47) = 3.49, *p* = .001, 95% CI [0.6, 2.3]. Target capture rates followed the same trend but were only marginally significant (Present: 37.9%; Absent: *M* = 36.2%), *t*(47) = 2.02, *p* = .050, 95% CI [0.0, 3.5]. These results indicate that the benefit of distractor presence did not specifically enhance guidance towards the target-stimulus shape, but just *away* from the distractor-feature.^1^

#### Non-target and distractor dwell times

These dwell times were recorded as the amount of time spent fixating on ‘incorrect’ items (i.e., non-targets or the distractor) that were fixated with the first eye movement. All eye movements were recorded in the duration between stimulus onset and participant key response. Distractor dwell times (*M* = 165.0 ms) were significantly shorter than non-target dwell times (*M* = 171.2 ms), *t*(47) = 2.73, *p* = .009, CI [1.6, 10.7], indicating that participants were able to ‘reject’ the distractor more quickly than the target-similar non-targets.

#### Target dwell times

The target dwell times were measured as the accumulated time the eyes were fixated on the target across all target fixations in a trial. Results revealed a facilitation of target identification by distractor presence: target dwell times were shorter on distractor-present trials (*M* = 472.5 ms) compared to distractor-absent trials (*M* = 478.4 ms), *t*(47) = 2.56, *p* = .013, 95% CI [1.3, 10.5]. This indicated that the presence of the distractor facilitated decisional processes related to target identification or execution of the response.

#### Early versus late effects

To gauge the contributions of early search-related processes and later post-search processes, two time windows were partitioned: from the start of the trial to the point in time the eyes first fixated on the target (target localisation times; early effects), and the time from the first fixation on the target to the manual response (response latency; late effects).

**Fig. 3:**
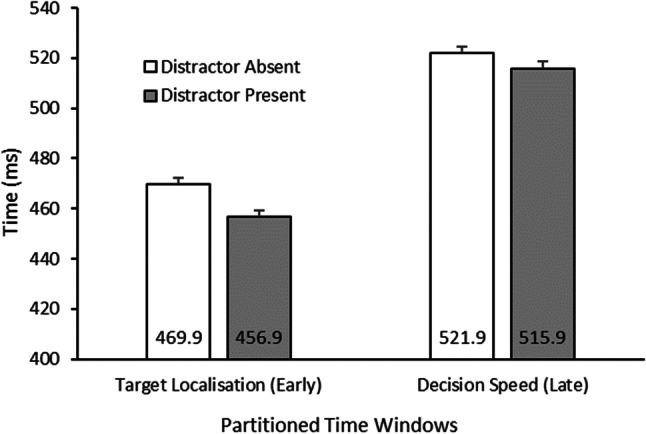
Breakdown of trial timings between when the target stimulus was first fixated and following latency before a keyboard response was made. Results revealed that the presence of the distractor led to both quicker localisations and decision resolutions

Target localisation times revealed that the target stimulus was fixated quicker in the distractor-present trials compared to the distractor absent trials, *t*(47) = 3.55, *p* = .001, 95% CI [5.6, 20.3], reflecting an early attentional facilitation of ~13 ms (see Fig. 3). Response latencies (i.e., time after attention has been guided to the target stimulus to the manual response) followed the same pattern. Distractor-present trials led to faster responses than absent trials, *t*(47) = 2.18, *p* = .034, 95% CI [0.5, 11.6], reflecting a late facilitation of responses of ~6 ms. Taken together, these results show that the overall beneficial effect of distractor presence (of 20 ms in the mean RT) are mainly (~65%) due to facilitation of early, attention-guiding processes and to a lesser extent (~30%) due to facilitation of later processes. However, the beneficial effects cannot be clearly attributed to inhibition of the distractor colour: As the target was always presented together with the distractor in the visual search trials, benefits could alternatively be due to tuning towards the target colour.

### Probe trials

As the probe task required participants to identify the numeral by pressing the corresponding numeric keys, RTs were quite long (*M* = ~2,100 ms) and did not show any differences between conditions. Hence, in the following we focus on eye movements and identification accuracy for the probe task.

#### Accuracy

**Fig. 4:**
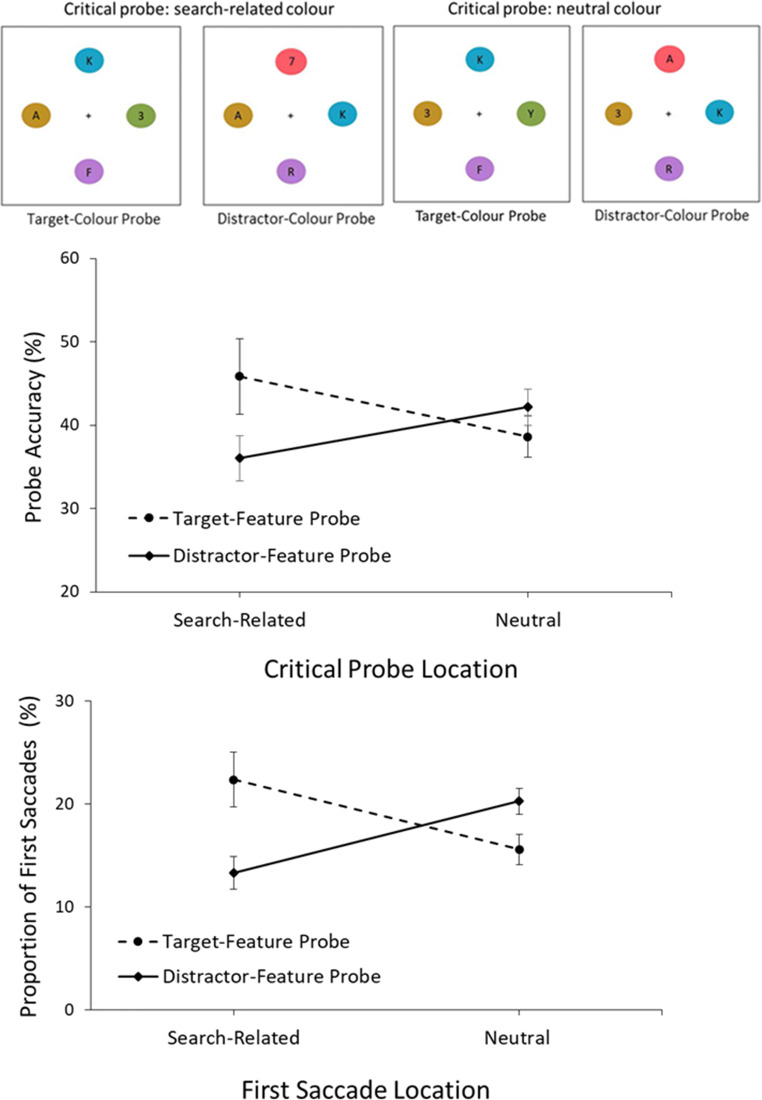
**Above:** Depictions of the four types of probe array, with the target colour depicted as green and distractor colour depicted as green in this example. **Below:** Results from the probe trials, with probe accuracy displayed on top, and first saccade location at the bottom. Results showed that on target-colour present probes, more first saccades went to the target-colour probe than to the neutral colours and that accuracy was higher when the critical probe was on the target-matching colour than on a neutral colour. Conversely on distractor-colour probe trials, the first saccade was *less* likely to be directed to the distractor-matching colour than to the neutral colours. Furthermore, accuracy was lower when the critical probe was on the distractor colour than when on a neutral colour, reflecting inhibition. Error bars represent within-subject 95% confidence intervals (Loftus & Masson, 1994)

Trials with early (< 100 ms) eye movements (4.5%) and trials with RTs longer than 5 s (1.1%) were excluded from analysis. A 2 (Probe Type: Target-colour present, Distractor-colour present) x 2 (Critical Probe Location: Search-related, Neutral) repeated-measures ANOVA was conducted on mean probe accuracy (as displayed in Fig. 4). No effects of probe type, *F*(1,47) = 3.59, *p* = .064, ƞ^2^_p_ = 0.07, or probe location were observed, *F*(1,47) = 0.08, *p* = .777. Importantly, the type x location interaction was significant *F*(1,47) = 11.38, *p* = .001, ƞ^2^_p_ = 0.20, reflecting that on target-colour probe trials, accuracy was higher when the critical probe was on the search-related colour (i.e., target colour; *M* = 46.0%) compared to the neutral colour (*M* = 38.8%), *t*(47) = 2.29, *p* = .026, 95% CI [0.8, 13.5]. This reflects enhancement of the target colour due to tuning to the target colour. On distractor-colour trials this effect was reversed: When the critical probe was on the distractor-colour probe, accuracy was lower (*M* = 36.2%) than when it was on the neutral colour (*M* = 42.4%), *t*(47) = 2.86, *p* = .006, 95% CI [1.8, 10.4], suggesting inhibition of the distractor feature.^2^

#### First eye movements

The probe identification results replicated the previous findings of Chang and Egeth (2019), demonstrating target facilitation and distractor inhibition. To probe early attentional processes, we next analysed eye movements on probe trials. Only trials where eye movements were made before the presentation of the probe characters were included to ensure that data reflected attentional biasing to the probe colours (19.5% of trials excluded). Trials where no eye movements were made were not included in this analysis (14.1% of trials excluded). Neutral probe fixations were measured the same way as the non-target fixations in visual search, i.e., the average number of first saccades directed towards a single neutral probe. Replicating the patterns seen in the accuracy data, on target-colour probe trials there was more capture by the target-coloured probe (*M* = 22.4%) compared to neutral probes (*M* = 15.6%), *t*(47) = 3.53, *p* = .001, 95% CI [3.5, 10.6]. The reverse effect was observed on distractor-related probes with distractor-coloured probes capturing fewer eye movements (*M* = 13.5%) than the neutral probes (*M* = 20.2%), *t*(47) = 6.01, *p* < .001, 95% CI [4.4, 8.9]. These effects establish that the beneficial effects of distractor presence observed in the search trials are in part due to inhibition of the distractor colour and the enhancement of the target feature and reveal that the effect is present at an early stage of visual processing (within the first eye movements). Comparing the relative contributions of target colour enhancement and distractor colour inhibition reveals that the distractor benefits are approximately equally due to target enhancement and suppression of the distractor (mean differences: 6.8% target colour enhancement vs. 6.7% distractor colour inhibition).^3^

## Discussion

It has become quite clear that the presence of a known distractor can facilitate search behaviour (Gaspelin et al., 2015), however, it has been unclear if these effects were due to distractor feature suppression (Arita et al., 2013) or target feature enhancement (Livingstone et al., 2017; Schönhammer et al., 2020). The current study helps to resolve this debate by using the more rigorous design of Chang and Egeth (2019) with the inclusion of eye-movement data to map the time course of inhibition and enhancement in more detail.

### Enhancement and suppression guide overt attention

In both visual search and probe trials an oculomotor suppression of the distractor feature was observed. In visual search, eye movements were less likely to be directed to the distractor item than to the non-targets, replicating previous studies (e.g., Gaspelin et al., 2017). While these results have regularly been attributed to inhibition of the distractor colour (e.g., Gaspelin et al., 2015), an alternative explanation was the exclusive up-weighting of the shared target and non-target colours. The probe trials allowed distinguishing between these explanations, as they contained only the target colour (among neutral colours) or only the distractor colour (among neutral colours). The results showed oculomotor suppression of the distractor colour even when it was presented with neutral colours, demonstrating distractor-inhibition independent from the potential target-enhancement.

The probe trials revealed that attention was guided not just by distractor suppression, but also by target enhancement. The target-coloured probe attracted eye movements over the neutral-coloured probes, indicating that attention was also biased towards the target colour (in addition to being biased away from the distractor colour). Even though the colour of the search target was shared with the non-targets, it would still be behaviourally beneficial to tune attention to this feature, as it creates a subset search that does not include the distractor (e.g., Egeth et al., 1984; Kaptein et al., 1995). These results support the mechanisms behind both inhibition theories (e.g. Treisman & Sato, 1990) and classical target-feature enhancement (Wolfe, 1994) as it seems that both attentional mechanisms work together to guide search behaviour (Chang & Egeth, 2019).

It should be noted that inferences were made about how attention was guided in visual search based on the biases observed in the probe trials. However, given that we measured early attentional biases (eye movements) in rare probe trials that were intermixed with the search trials, it is unlikely that separate guidance strategies were in place for probe and search trials. The biases observed in the probe trials are likely to be representative of how early attention was guided in search.

### Time course of distractor benefits

In addition to distinguishing between target-feature enhancement and distractor-feature suppression, oculomotor data were used to assess the time-course of distractor benefits on target localisation. When the distractor was present on search trials, eye movements landed on the target item sooner than when the distractor was absent. This was combined with a weak effect suggesting that the first eye movement was more likely to be directed towards the target on distractor-present than distractor-absent trials. Together this implies that the distractor presence led to early attentional benefits for locating that target item. This is consistent with previous studies that identified event-related potential components suggesting early impacts of inhibition (Gaspelin & Luck, 2018; Ipata et al., 2006; Moher et al., 2014; but see Drisdelle & Eimer, 2021; Kerzel & Burra, 2020; Livingstone et al., 2017). These novel, target localisation effects of distractor presence may only be able to be observed under specific visual search arrays with favourable circumstances (e.g., low set size or the relatively large distance between stimuli used in the current design), as previous studies using similar paradigms have failed to observe target localisation benefits (Gaspelin et al., 2017). For example, Gaspelin et al. (2017) found inhibition with using a set size of six items (compared to the current four items), but no evidence for a target-localisation benefit. Potentially, the additional non-target search stimuli diffused attention, leading to more non-target selections, rendering it difficult to detect the target-localisation benefit in the RT and latency-based measures.

Previous inhibition studies in the visual search paradigm have used the RT facilitation effects as an indicator of how the distractor helped to guide attention. In the current design, we revealed that this facilitation effect was not just composed of an early localisation benefit. After participants had located the target item, they were also quicker to make a key response to the target when the distractor was present rather than absent, though this effect was numerically smaller than the early localisation benefit (~6 ms compared to ~14 ms). Late response benefits could potentially be due to higher confidence levels (or reduced response thresholds) after localising the target, as one of the items had been eliminated as a potential target (i.e., the distractor). An alternative proposal is that the more dynamic colour configuration in the probe displays led to higher arousal and thus speeded responses (e.g., Lundqvist et al., 2014).

Moreover, distractor presence led to clear search benefits across intermediate stages in visual search. Distractor dwell times were shorter than non-target dwell times, and target dwell times (before a response was made) were shorter in the presence of a distractor. Together these results suggest that previous observations of inhibition effects were a combination of both early attentional processes and processes at a later stage (e.g., Gaspelin et al., 2015). These results contribute to the growing body of research that suggests caution when making inferences about early attentional processes based upon RT and accuracy data (Hamblin-Frohman & Becker, 2021).

It should be noted that attention and eye movements are not perfectly correlated. Eye movements and attention are intimately coupled, in that covert attention needs to shift to the saccade target location prior to the eye movement (e.g., Deubel & Schneider, 1996). However, while we are fixating, covert attention can shift to other locations (Posner, 1980). It is therefore possible that the distractor is selected covertly and is then followed by rapid disengagement of attention (e.g., Theeuwes et al., 2000). A rapid disengagement account (Sauter et al., 2021) is still unlikely to fully account for our results, as covert attention shifts are time-consuming and covert selection of the distractor should thus delay eye movements to the target (as covert attention first needs to shift to the target location before an eye movement can be executed; Deubel & Schneider, 1996). By contrast, we found that distractor presence *speeded* target-localisation latencies (~13 ms) and thus our results do not show evidence for rapid disengagement, yet does not provide strong evidence against this theory.

Relatedly, it has been proposed that inhibition can only be observed when participants perform a serial search or a clump-wise serial search, where the search items are scanned systematically until the target is found (e.g., Liesefeld & Müller, 2020; Wang & Theeuwes, 2020). While this explanation might be applicable to studies measuring RT and errors, the present eye-movement measures render this unlikely. A serial or inefficient search is characterised by no selectivity in the first eye movements, viz., equal selection of all search items, with distractor skipping occurring only in later fixations (e.g., Horstmann et al., 2017). By contrast, our results showed clear evidence that target enhancement and distractor inhibition already influenced the first eye movement. First eye-movement data are usually taken as evidence that attention was biased to (or against) specific items prior to the onset of the search array (e.g., Becker et al., 2017; Failing et al., 2015; Ludwig & Gilchrist, 2003; Martin & Becker, 2018).

In addition, our results are also not consistent with the proposal that we reactively inhibit saliency signals (Sawaki & Luck, 2010). According to Sawaki and Luck (2010), salient items produce an automatic attend-to-me signal that can be quickly inhibited (below baseline) when salient items are known to be irrelevant. By contrast, we found clear evidence for inhibition in the probe trials, in which the distractor colour was not salient. This shows that items do not need to be salient or evoke an *attend to me* signal to be suppressed. Rather, attention was guided away from the distractor colour via inhibition of the distractor feature value (i.e., the specific colour of the distractor; e.g., Treisman & Sato, 1990; see also Ruthruff et al., 2021), not via reactive inhibition of saliency signals (e.g., Sawaki & Luck, 2010). With this, our results are in line with the results of Lien et al. (2021), who showed that suppression effects are equivalent between search arrays containing a single (thus salient) distractor and multiple distractors. Indeed, such results are consistent with findings from an older literature on visual search (e.g., Becker & Horstmann, 2009; Egeth et al., 1984; Kaptein et al., 1995; Treisman & Sato, 1990) in which participants were found to be able to limit selection to a subset of items in a conjunction search task (e.g., selecting only red items in search for a red, tilted item among red vertical and green tilted items).

## Conclusion

The current experiment provides a more complete picture of how distractor suppression is incorporated into visual search strategies. We extended the research of Chang and Egeth (2019) by assessing the differential effects of target enhancement and distractor suppression with the more direct measurement of eye movements, which allowed (1) an estimate of the relative contributions of both effects, and (2) separate evaluation of the contributions of early, intermediate and late processes. Both the previous and the current study show that the typical inhibition effect is a combination of true inhibition of the distractor feature value and enhancement of the target feature, which both guide visual search. As a novel contribution, we reveal that these biasing effects have benefits at both an early stage of attention, helping to locate the target stimulus, and at a later stage, speeding the response to the target. These two influences combine to create the RT benefit observed in previous inhibition studies and suggest that attentional biasing can utilise both enhancement and suppression.

## Footnotes

1 We also inspected the latencies of first fixations on each item, which showed the typical results patterns reported in previous studies: On distractor-present trials, the distractor fixation latencies were shortest (204.6 ms), followed by non-target fixation latencies (211.3 ms), while target fixation latencies were longest (236.8 ms). All of these latencies differed significantly from each other, all *p*s < .05. Distractor present vs. absent trials did not differ in terms of the latencies (non-target: 211.3 ms vs. 211.2 ms; *n.s.*; target: 236.7 vs. 236.8 ms; *n.s.*).

2 We also compared accuracies for distractor-colour-present probe trials that were proceeded by a distractor-present or distractor-absent search trial and found no differences (both when the critical probe was on the distractor colour or a neutral colour), *ts*(47) < 1.2, *p*s > .236. This suggests that the bias away from the distractor colour was not due to a short-term suppression and carry-over effect, but to a more long-lasting form of inibition.

3 We also inspected the latencies of first fixations to any items on the target vs. distractor-coloured probes on probe trials. The latencies did not differ, with latencies of 218 ms to target-coloured probes, and latencies of 214 ms recorded for distractor-coloured probes, *p* = .418.
